# A febrile papulovesicular rash in a patient with pyoderma gangrenosum

**DOI:** 10.1111/ddg.15887

**Published:** 2025-09-21

**Authors:** Anika Rajput Khokhar, Charlotte S. Wilm, Katharina Meier, Kamran Ghoreschi, Ulrike Blume‐Peytavi, Farzan Solimani

**Affiliations:** ^1^ Department of Dermatology Venereology and Allergology Charité – Universitätsmedizin Berlin corporate member of Freie Universität Berlin and Humboldt‐Universität zu Berlin Berlin Germany; ^2^ Fachklinik Hornheide Münster Germany

Dear Editors,

Pyoderma gangrenosum (PG) is a rare autoinflammatory neutrophilic dermatosis characterized by rapidly growing, highly painful ulcerations, most commonly on the lower extremities. PG treatment targets the reduction of disease activity and healing of ulcers. Therapeutic concepts are based on various immunosuppressive drugs, including systemic corticosteroids, ciclosporin, and tumor necrosis factor α inhibitors.^1^, ^2^ The need for long‐term immunosuppressive treatment and the presence of large wounds place this patient group at increased risk of developing infectious diseases.^3^ Immunocompromised patients require special instruction and care regarding vaccination and reactions to infections, since *(1)* infections can show a more severe course due to the immunocompromised status and *(2)* infections might worsen the underlying inflammatory or autoimmune condition. Therefore, precautions including preventive measures (e.g., vaccination) are a pivotal step for the management of this group of patients.

A recent case from our clinic illustrates this concept. A 21‐year‐old female patient with a history of PG was admitted with fever and a new polymorphic exanthema, characterized by small erythematous translucent vesicles interspersed with necrotic papules, predominantly on the face, upper body, and genital area, accompanied by oral aphthae (Figure 1). Her symptoms had intensified over the past 3 days, along with severe lower back pain. The patient was originally from Ukraine where PG was first diagnosed. She was receiving immunosuppressive therapy with cyclosporine (300 mg/day) in combination with prednisone (10 mg/day). Her medical history was negative for chickenpox. She did not recall having received childhood vaccinations and had no vaccination record. The patient was hospitalized for further diagnostic and therapy.

**FIGURE 1:**
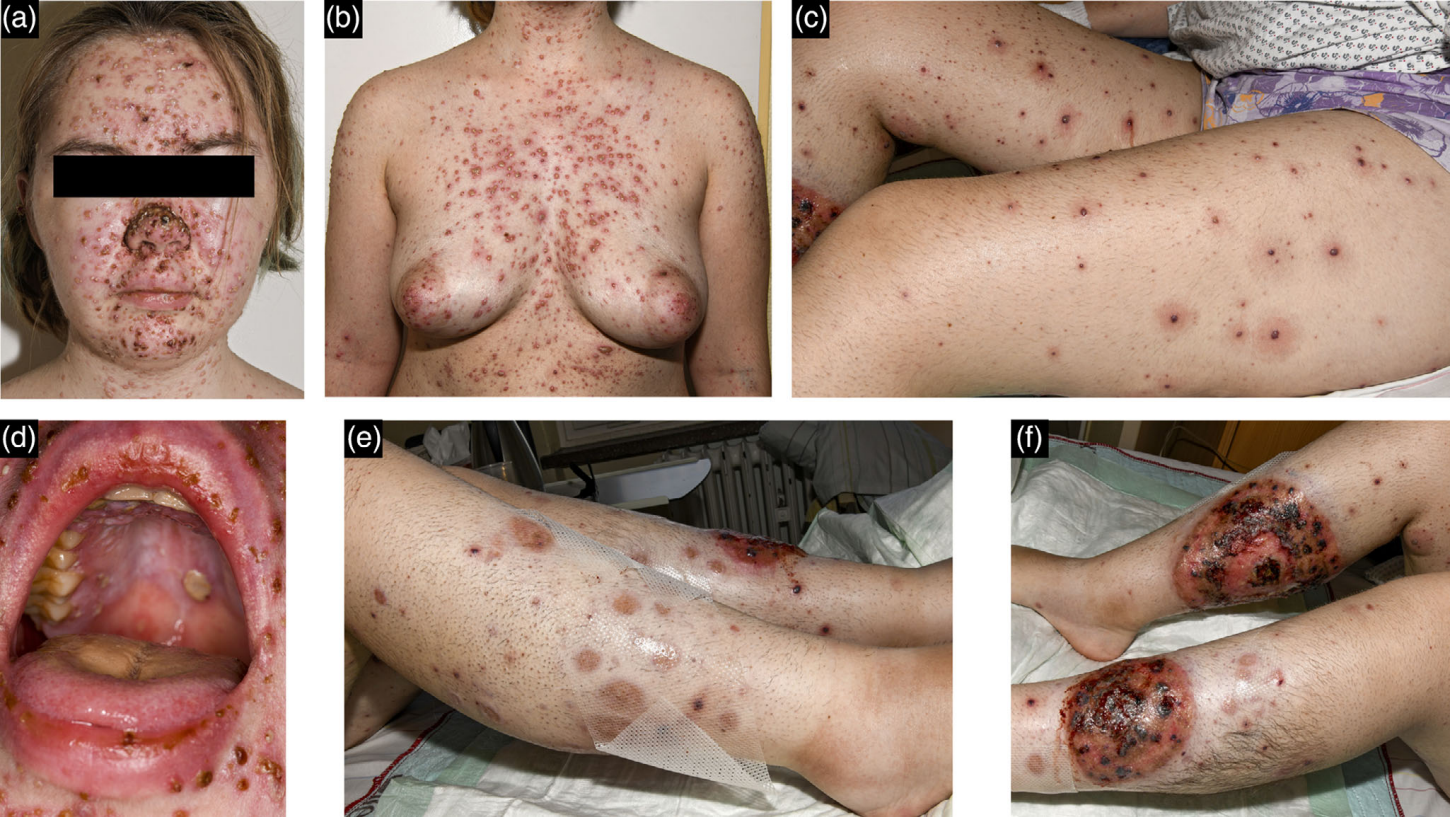
(a–d) Clinical presentation of infection with varicella zoster virus and (e–f) parainfectious worsening of pyoderma gangrenosum.

Suspecting a primary infection with varicella zoster virus (VZV) under iatrogenic immunosuppression, antiviral therapy with acyclovir (10 mg/kg body weight [BW]) was initiated and cyclosporine was paused. VZV infection was confirmed by polymerase chain reaction (PCR) of a cutaneous swab and serological detection of IgM and IgG antibodies. Measles antibodies were not detectable.

Due to markedly elevated inflammatory markers – C‐reactive protein 259.3 mg/l (reference range < 5 mg/l) and leukocytes 13.88/nl (reference range 3.90–10.50/nl) – we initiated broad‐spectrum antibiotic therapy with piperacillin–tazobactam (4.5 g every 8 hours). Infectious diseases such as hepatitis B/C, tuberculosis and HIV infections were excluded. Since adults with VZV may develop pulmonary involvement, we performed a chest X‐ray, which ruled out VZV pneumonia. Urinalysis and blood cultures did not reveal additional infections. During the VZV infection and the drug‐free interval, her PG deteriorated rapidly with respect to pain, size, and number of ulcerative lesions. Because of the concurrent VZV infection, we refrained from immunosuppressive therapy and initiated intravenous immunoglobulin (2 g/kg BW over 5 days) for the PG exacerbation.^4^ After completion of antiviral therapy and clinical control of the VZV infection, we switched the immunosuppressive regimen to oral prednisone (50 mg daily) and infliximab (5 mg/kg BW). Additionally, new joint pain and swelling were noted, with positive anti–citrullinated protein antibody (ACPA, 26.2 U/ml [reference range < 20 U/ml]) but no elevation in rheumatoid factor or HLA‐B27 positivity. In consultation with our rheumatologists, we suspected a reactive arthritis and initiated methotrexate (10 mg subcutaneous [SC]) to taper prednisone and avoid the chronic use of high doses of steroids. Multidisciplinary care included pain management and psychosomatic support.

This case of varicella infection in a young patient with PG highlights the necessity of an up‐to‐date vaccination status prior to initiation of immunosuppressive therapies. Since 2004, the *German Standing Committee on Vaccination* (STIKO) has recommended varicella vaccination for all children up to 18 years of age and for adults with specific indications, including seronegative individuals before planned immunosuppressive therapy or organ transplantation. However, recommendations for varicella vaccination differ globally and even within the European Union. The WHO recommends this vaccination only in countries where resources are sufficient to achieve and maintain coverage ≥ 80%, as lower rates may shift infections to older age groups, thereby increasing morbidity and mortality. In Ukraine, VZV vaccination is not recommended, while in Poland it is recommended only for specific at‐risk groups. By contrast, varicella vaccination is generally recommended in countries such as France, Greece, and Luxembourg, and is even mandatory in Hungary and Italy.^5^ Moreover, in immunosuppressed patients with VZV seropositivity or after varicella vaccination, shingles vaccination should be considered. The former is a non‐live recombinant vaccine that can be administered during immunosuppressive therapy, whereas the VZV vaccine is a live attenuated vaccine and must be given with an appropriate safety interval before initiating therapy.

In our case, factors such as loss of the vaccination certificate, language barriers, and limited access to medical care, including a general practitioner, resulted in a suboptimal vaccination status during immunosuppressive treatment and a severe varicella infection. Enhanced efforts, such as internationally standardized childhood vaccination schedules, are needed to safeguard this high‐risk group.

## CONFLICT OF INTEREST STATEMENT

None.

## References

[1] Maronese CA , Pimentel MA , Li MM , et al. Pyoderma Gangrenosum: An Updated Literature Review on Established and Emerging Pharmacological Treatments. Am J Clin Dermatol. 2022;23:61534. 10.1007/s40257-022-00699-8 PMC9464730 35606650

[2] Zaman M , Martinez R , Mayur O , et al. Use of biologic therapies in the management of pyoderma gangrenosum: a systematic review. Arch Dermatol Res. 2024;316:539. 39158753 10.1007/s00403-024-03332-2

[3] Maverakis E , Marzano AV , Le ST , et al. Pyoderma gangrenosum. Nat Rev Dis Primers. 2020;6:81. 33033263 10.1038/s41572-020-0213-x

[4] Enk A , Hadaschik E , Eming R , et al. Europaische Leitlinien (S1) fur die Anwendung von hochdosierten intravenosen Immunglobulinen in der Dermatologie. J Dtsch Dermatol Ges. 2017;15:227‐238. 28214313 10.1111/ddg.13013_g

[5] Stoevesandt J , Schmalzing M , Mohme S , Goebeler M . Vaccination in dermatology 2025: update considering current recommendations of the German Standing Committee on Vaccination. J Dtsch Dermatol Ges. 2025;23(8):925‐930. 40495641 10.1111/ddg.15785 PMC12338432

